# Development of an Improved QCM-D Instrumentation for Affinity Sensing by Bioinspired Molecular-Imprinted Polymers (MIP) for IgG Detection in Serum

**DOI:** 10.3390/s26102985

**Published:** 2026-05-09

**Authors:** Doretta Cuffaro, Lucia Bonasera, Elisa Nuti, Riccardo Galletti, Manuela Adami, Marco Sartore, Maria Minunni

**Affiliations:** 1 Department of Pharmacy, University of Pisa, 56125 Pisa, Italy; lucia.bonasera@edu.unito.it (L.B.); elisa.nuti@unipi.it (E.N.); 2 ElbaTech Srl, 57030 Marciana, Italy; galletti@elbatech.com (R.G.); adami@elbatech.com (M.A.); sartore@elbaetch.com (M.S.)

**Keywords:** quartz crystal microbalance (QCM), molecularly imprinted polymers (MIPs), polynorepinephrine (PNE), Human Immunoglobulin G (hIgG), clinical diagnostics

## Abstract

Quartz crystal microbalance (QCM) technology provides a powerful, label-free platform for monitoring molecular interactions in real time with nanogram sensitivity. Recent advances in compact instrumentation have enhanced analytical performance while reducing energy consumption, aligning with the principles of Green Analytical Chemistry. In parallel, the European Union has recommended the replacement of animal-derived antibodies with non-animal alternatives, creating an urgent need for sustainable affinity receptors. In this study, we present an innovative application of polynorepinephrine (PNE)-based molecularly imprinted polymers (MIPs) with a compact QCM sensing. PNE, a bioinspired polymer formed under mild aqueous conditions, offers strong adhesive properties and biocompatibility, enabling robust immobilization of imprinted receptors on gold-coated quartz disks. The resulting PNE-MIP/QCM platform combines the ultrasensitivity of quartz microbalances with the selectivity of molecular imprinting, delivering a reproducible and environmentally responsible affinity sensor. The sensor showed a limit of detection of 11.2 nM and enabled accurate IgG quantification in diluted human serum samples. As a proof of concept, the system was applied to Human Immunoglobulin G (IgG1) detection, demonstrating its potential for sustainable clinical diagnostics.

## 1. Introduction

Quartz crystal microbalance (QCM) technology has established itself as a highly sensitive and versatile platform for affinity sensing, capable of detecting minute mass changes at the sensor surface in real time and without the need for labels. The studies on piezoelectric transduction in analytical chemistry started long ago [1,2,3,4,5,6,7]. We eventually began in the early nineties to apply QCM transduction to affinity sensing, moving initially from the use of antibodies as natural receptors to nucleic acids as molecular probes for the development of hybridization-driven DNA sensing, and more recently to biomimetic receptors such as aptamers and molecularly imprinted polymers, thus following for over thirty years the evolution and the emerging trends in affinity-based sensor development. What still attracts the interest of researchers in developing piezoelectric sensing and instrumentation is the ability of the QCM to provide both qualitative and quantitative information, through frequency shifts proportional to mass variations, and qualitative insights, via dissipation monitoring of viscoelastic properties; these features make QCM attractive for affinity sensing, where ligand–analyte binding can be followed directly without secondary markers. Quantitative information is obtained from calibration curve since the frequency shifts are proportional to mass variations according to the Sauerbrey equation in gas phase [8], while in liquid media the Kanazawa equation introduces viscosity and density terms to account for viscoelastic effects. In addition, dissipation monitoring (QCM-D) yields insights into the viscoelastic properties of adsorbed layers, enabling the study of conformational changes and rigidity of biomolecular assemblies. A recent comprehensive review reports principles and application of QCM-based bioanalytical assays [9]. Overall, the quartz crystal microbalance (QCM) represents an environmentally friendly analytical technique in the context of Green Analytical Chemistry. Its label-free detection principle eliminates the need for dyes, radioactive tracers, or fluorescent labels, thereby minimizing the use of hazardous chemicals and auxiliary reagents. Owing to its extremely high mass sensitivity, QCM measurements can be performed using very small amounts of analyte, which reduces material consumption and experimental waste. In addition, QCM enables real-time and in situ monitoring of interfacial processes, avoiding labor-intensive sample preparation steps such as extraction, separation or derivatization. The instrumentation operates at low energy consumption, without the need for high temperatures or vacuum systems. Furthermore, quartz crystal sensors can often be regenerated and reused multiple times, contributing to waste reduction over the instrument’s life cycle. Overall, QCM constitutes a sustainable alternative to more resource-intensive analytical techniques, aligning with key principles of Green Analytical Chemistry (GAC), including waste prevention, reduced use of solvents and auxiliaries, and real-time monitoring for pollution prevention [10].

The development of compact QCM instrumentation represents a significant advancement [11], combining high sensitivity with operational simplicity and reduced energy consumption in alignment with the principles of GAC. Building on these foundations, we designed the WinQCM apparatus, a compact and portable device. WinQCM allows quantitative investigation of both mass loading effects and viscoelastic responses, while maintaining a low-cost architecture coupled with high analytical performance, achieving frequency resolution in the sub-Hertz range. The instrument is characterized by low intrinsic noise (RMS frequency noise: 0.05 Hz @ τ = 1 s) and provides data output compatible with the most widely used spreadsheet and data analysis software. WinQCM is an environmentally sustainable analytical instrument, modular and customizable to accommodate specific experimental protocols and user-defined requirements, suitable for laboratories engaged in bioanalysis as well as in the study of adhesion processes and intermolecular interactions among chemical and biochemical species.

In this paper we combine WinQCM instrumentation with innovative and sustainable affinity receptors in line with the 2020 Recommendation of the EU Reference Laboratory for Alternatives to Animal Testing (EURL ECVAM), issued in May 2020 by the European Commission’s Joint Research Centre (JRC); the recommendation states that animals should no longer be used for the development and production of antibodies (Abs) for research, diagnostics, and regulatory applications [12].

Scientifically valid alternatives to Abs exist, including recombinant antibodies based [13,14,15,16,17] on modern in vitro selection technologies (e.g., phage display) providing reagents with equal or superior specificity, reproducibility, and performance; aptamers are obtained through in vitro selection processes, while molecularly imprinted polymers (MIPs), produced by chemical synthesis, have demonstrated the capability to replace antibodies in bioanalysis, opening new the perspective of animal-free affinity receptors. A recent review [18] describes aptamer-based QCM sensing analysis techniques and applications. MIP-QCM sensors have been reported achieving detection limits ranging from sub-nanomolar to low microgram per liter concentrations for a wide variety of analytes, from small molecules (pesticides, antibiotics, mycotoxins) to large biomolecules (proteins, immunoglobulins) [19]. Most of the monomers used are acrylamides, and the MIP polymer synthesis occurs in organic solvents, eventually producing toxic byproducts. More recently, the need to improve the sustainability of MIP synthesis and production has become increasingly evident [20], prompting the scientific community to explore the use of naturally derived monomers for the imprinting process [21].

Our group has been active for over a decade in developing MIPs derived from neurotransmitters (catecholamines) [22,23,24,25] which offer the advantage of undergoing spontaneous auto polymerization in water under basic conditions on a variety of surfaces, while exhibiting intrinsic adhesive properties. This represents a significant benefit, as the resulting biomimetic MIP receptor does not require additional immobilization or functionalization steps to anchor it onto the transducer surface, thereby avoiding the use of potentially hazardous chemical agents.

Polynorepinephrine (PNE) [23,25] is a bioinspired polymer obtained from the spontaneous oxidation in water under mild alkaline conditions of the catecholamine norepinephrine (NE). PNE presents some advantages such as material-independent adhesion—the coating forms on a remarkably broad range of substrates, reducing the use of reagents, including potentially toxic organic solvents—and simplicity; the process requires only immersion in NE solution at slightly basic pH in the presence of the target to be imprinted. As a proof of concept, we targeted the quantitative analysis of Human Immunoglobulin G (hIgG), with particular focus on the IgG1 subclass. MIP-based QCM sensing has been previously reported using methacrylic acid (MAA) as the monomer for bulk polymerization in the presence of ethylene glycol dimethacrylate (EGDMA) as cross-linker and IgG as the template; poly(methacrylic acid) (PMAA) achieved IgG detection in the range 1–5 ng/mL in buffer [26].

IgG1 is clinically relevant in allergy, autoimmune disorders, hemolytic disease of the newborn, and immunotherapy. Antibody deficiencies represent the most common form of immunodeficiency in patients with impaired immune function, making the determination of immunoglobulin levels highly important. Human IgG is divided into four subclasses, of which IgG1 was selected as the target in this study. In allergic individuals, allergen-specific antibodies are predominantly of the IgG1 and IgG4 subclasses, while IgG2 and IgG3 responses are comparatively minor. Reference values established by the WHO serum [19] indicate concentrations of 5.0 g/L for IgG1, 2.6 g/L for IgG2, 0.4 g/L for IgG3, and 0.5 g/L for IgG4 [20], which have been endorsed by the International Union of Immunological Societies (IUIS) as the correct standard. During most infections, IgM antibodies appear first, followed by IgG antibodies that provide long-term immunological memory of the pathogen. Moreover, IgG1 plays a central role in autoimmune diseases, where autoantibodies are frequently of this subclass, and is critically involved in severe cases of hemolytic disease of the newborn [27].

The aim of this work is to develop a sustainable affinity-based sensing platform built on a newly engineered compact QCM instrument (WinQCM), combined with bioinspired PNE molecularly imprinted polymers (PNE-BMIP) for IgG detection. PNE-MIPs targeting IgG had already been established by our group and successfully applied to optical sensing for the quantitative analysis of IgG in human serum [24,25]. In the present study, these materials are further adapted and integrated into a QCM interface to enable a more sustainable and versatile sensing approach.

In this context, we combine for the first time a sustainable PNE-MIP, targeting hIgG in serum, with label-free, real-time QCM sensing using WinQCM instrumentation into an innovative sensing platform. This approach exploits the advantages of PNE imprinting and acoustic transduction to deliver a compact, low-energy, and environmentally conscious platform for affinity sensing, aligned with the principles of Green Analytical Chemistry, broadening the range of platforms compatible with self-polymerized PNE green MIP.

## 2. Materials and Methods

### 2.1. Chemicals

L-norepinephrine chlorhydrate (NE, ≥98.0%), Tris(hydroxymethyl)aminomethane hydrochloride, (Tris-HCl, ≥99.0%), sodium dodecyl sulfate (SDS, ≥98.5%), sodium chloride (NaCl, ≥99.0%), sodium hydroxide (NaOH), hydrochloric acid (HCl), glacial acetic acid (≥99.7%), 6-mercapto-1-hexanol (MCH, 97.0%), 11-mercapto-1-undecanol (MCU, 97.0%), ethanol (EtOH), sodium hypochlorite solution (6–14% active chlorine) and sodium azide were purchased from Merck (Darmstadt, Germany). The peptide 439KSLSLSPGK447 (acronym Fc CH3, MW = 916.08 g mol^−1^, purity > 95%) belongs to the heavy chain of the IgG1 constant domain, was used as a template peptide and was purchased from GenScript (Leiden, The Netherlands). Human serum albumin (HSA), bovine IgG and human serum from human male plasma AB were purchased from Merck (Darmstadt, Germany).

Buffer solutions: Milli-Q™ ultrapure water (R ≥ 18.2 MΩ cm), hereafter referred to as water, was used for the preparation of buffer solutions and during all synthesis procedures. TRIS-HCl buffer (10 and 50 mM Tris-HCl pH 8.5) was used for the synthesis of the PNE polymer film. Phosphate-buffered saline (PBS) was prepared from tablets containing 140 mM NaCl, 10 mM phosphate buffer and 3 mM KCl, pH 7.4 at 25 °C purchased from Merck (Darmstadt, Germany) and used for WinQCM assays, 15 mM sodium azide was added to the PBS buffer. All buffer solutions were then filtered through a 0.22 μm Millipore™ microporous filter (Merck, Darmstadt, Germany).

Equipment: For the synthesis of PNE-MIP films, a FOC 120I Connect incubator (VELP Scientifica, Usmate (Monza) Italy) was used. For UV-Vis absorption measurements, 96-well microtiter plates (JET BIOFIL Alicante, Spain, and Corning^®^, Glendadale, AZ, USA) were used. For UV-Vis absorption spectrophotometry measurements, the SPECTROstar Nano, a 96-well microtiter plate reader produced by BMG Labtech (Ortenberg, Germany), was used. The instrument measures the UV-Vis absorbance in the range between 220 and 1000 nm, as a single wavelength or as a spectrum, with a maximum resolution of 1 nm. QCM sensor chips: 10 MHz AT cut, QCM quartz crystals, with 13.95 with 6.5 mm for the quartz and electrode diameters, respectively, were purchased by I.E.V. Industria Elettronica Varese (Induno Olona (Varese), Italy).

### 2.2. Quartz Crystal Microbalance Gravimetric Sensing by WinQCM Apparatus

All piezoelectric transduction experiments using the quartz microbalance (QCM) were carried out using the WinQCM apparatus, a very compact portable instrument, currently in prototype form, produced and kindly made available by ElbaTech Srl (Marciana (LI), Italy), at the Department of Pharmacy, University of Pisa.

#### 2.2.1. WinQCM Electronics

**Figure 1:**
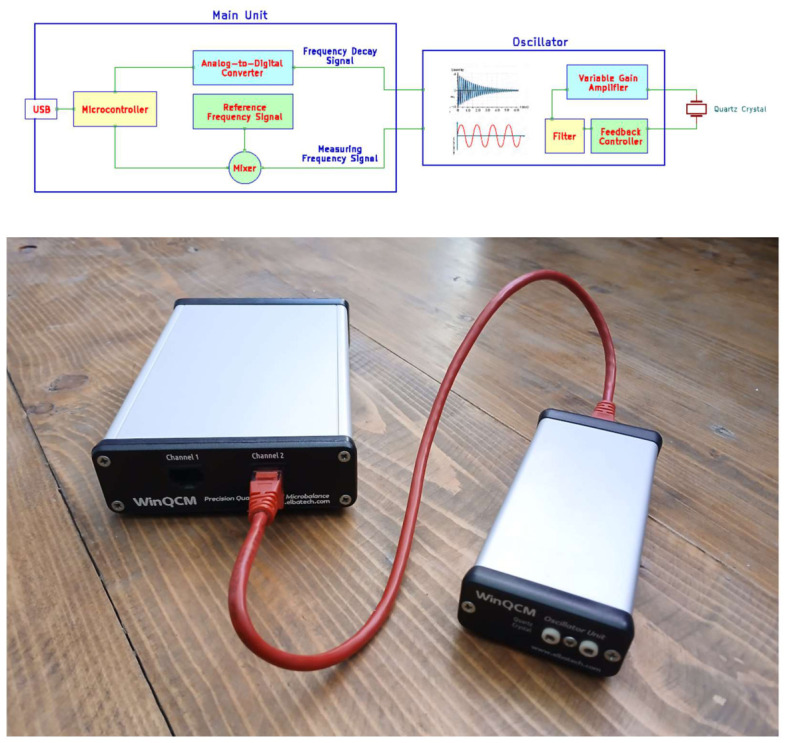
Schematic diagram of the WinQCM instrument. (**Top**) The oscillator includes a feedback controller to sustain the quartz crystal oscillation even when heavily loaded. It is actuated by a VGA (Variable Gain Amplifier) with the quartz directly part of the feedback loop. The main unit’s hearth is an STM32 Micro Controller programmed to serve both as an ADC bridge to acquire the analog damping signal and as a frequency counter. (**Bottom**): Picture of the WinQCM Instrument, with the small oscillator enclosure on the right and the main unit on the left.

The WinQCM electronics is composed of two main building blocks as usual for any QCM device, but equipped with added features: the oscillator and the reading units (Figure 1). Regarding the former, many circuits are known for simple operations, such as running a quartz crystal at its fundamental frequency. All these solutions are based on a signal amplifier providing “positive feedback” such that the output signal is fed back at the input with 180° phase rotation (Figure S1A), thus causing the amplifier to move to saturation and yielding to a stable oscillation.

These oscillators are very stable as long as the quartz crystal is close to its ideal equivalent circuit (Figure S1A), essentially when it is well protected from external interactions. That is why normal oscillators in electronics, as clock generators, make use of well protected, vacuum-encapsulated quartz crystals. But, the main feature of a QCM being the detection of a mass change at the crystal’s surface, the quartz element is inherently exposed to the environment and possibly to heavy mass loads. In this case the Quality Factor Q of the quartz strongly decreases, and the oscillation frequency can lead to feedback phase shifts far away from the ideal 180°, thus preventing the oscillator from working under these not-ideal conditions, which are the standard conditions during a QCM experiment. This problem calls for more sophisticated oscillator circuits.

The WinQCM oscillator derives from and is an improvement of a project of the Rowland Institute at Harward called RIS-727, designed in collaboration with Prof. Winfield Hill, head of the Electronics Department of that Institute [28]. The RIS-727, in turn, was inspired by the solution adopted by Stanford Research Systems (SRS) in their QCM25 oscillator (https://www.thinksrs.com/downloads/pdfs/manuals/QCM200m.pdf, accessed 16 March 2026).

The idea beyond these and our circuit is to add to the basic oscillator a true feedback controller capable of detecting excessive phase changes and promptly correct them, providing to an Automatic Gain Controller amplifier (AGC model AD8367) (https://www.analog.com/media/en/technical-documentation/data-sheets/AD8367.pdf accessed 16 March 2026) the desired 180° phased-out signal, thus sustaining the oscillation also under heavy load conditions at the crystal surface.

The WinQCM oscillator adds two more features: it can work at superior harmonics (feature not exploited in the present work) and can toggle the ON–OFF feedback loop while monitoring (during the OFF state) the oscillation decay. The latter feature allows us to measure the decay time constant, which is a direct measure of the quartz Quality Factor. Moreover, this “ring-down” method is very quick, thus allowing us to maintain a high data acquisition rate, which is of main importance in all those experiments in which following a kinetic of some type is the target goal. Thanks also to an optimized frequency reading system, the WinQCM can acquire a pair of f-Q readings every 0.1 s.

The quartz sensor is electrically decoupled from the system ground by a pair of RF Transformers (Figure S1B). This topology enables electrochemical experiments (such as potentiometric, etc.) to be carried out in parallel with the QCM measurements, without affecting their quality.

The system is based on the ST-Microelectronics STM32F401 microcontroller (Geneva, Switzerland) (https://www.st.com/resource/en/reference_manual/rm0368-stm32f401xbc-and-stm32f401xde-advanced-armbased-32bit-mcus-stmicroelectronics.pdf accessed 16 March 2026), a powerful programmable unit equipped with dedicated modules capable of working autonomously, i.e., freeing the Central Processing Unit (CPU) from taking care of device-specific tasks. Timers and communication hardware are examples of such dedicated modules.

The readout electronics is wired to the oscillator, that can be then located slightly far away from the former, for example, in a temperature-controlled chamber. To allow fast and precise readouts, it exploits a “frequency mixer” technology, where the quartz signal (*f_osc_*)is mixed with a precise and thermally stable local frequency signal (*f_ref_*) and then low-pass filtered to obtain their difference (*f_mix_*).

To retrieve the original quartz oscillation frequency with sub-Hz resolution, a sophisticated method is implemented (Figure S1C): a hardware Timer programmed to operate as a so-called “Output Compare” module provides a trigger signal every time it counts N_mix_ pulses of signal *f_mix_*. The time elapsed between two such consecutive trigger signals (*T_count_*) is then utilized as the “gate interval” for a second Timer programmed to work as a so-called “Input Capture” module: it counts the number of pulses of a very high-frequency clock signal, in our case the system clock (84 MHz), that occurred within a *T_count_* gate interval. Being the latter operation based on a very fast signal, the overall frequency measuring resolution referred to the quartz signal is as low as 10^−2^ Hz for typical QCM crystals of 5-to-20 MHz fundamental frequency.

The ring-down signal related to the Quality Factor measurement is instead an analog signal with exponential decay; therefore, it is acquired by means of a 12-bit Analog-To-Digital converter (ADC) embedded into the microcontroller unit. To synchronize the acquisition exactly with the feedback-loop OFF state, the ON–OFF signal to the oscillator stage is generated by the CPU itself, which works as a kind of “master” with respect to all the embedded and external modules.

#### 2.2.2. WinQCM Measuring Chamber

**Figure 2:**
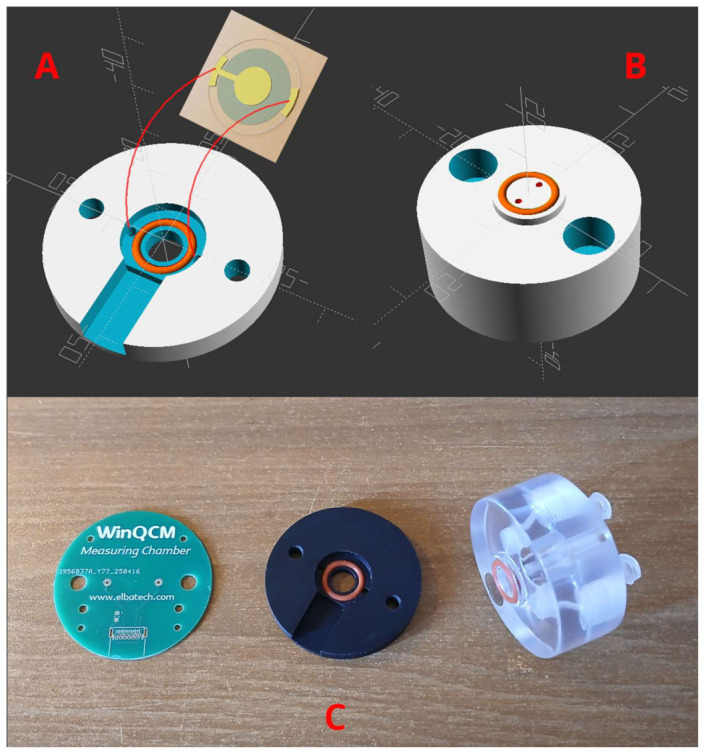
Schematic 3D drawing of the chamber base (part (**A**)) and top (part (**B**)). The red arcs show the quartz electrodes positioning in the cell. A Printed Circuit Board (PCB) located underneath has spring contacts matching the two little holes. The other side of the sensor (visible in transparency) is then contact-free and can face both a “static” and a flow-through top chamber. The latter is visible in part (**B**,**C**)) from left to right: the un-populated PCB with pads for the spring contacts, the base with inner quartz holder, the top flow-through part. The latter is realized in transparent material, chemically compatible, to allow for a fast visual inspection (for example to check immediately if any bubble is formed along the fluid channel from inlet to outlet).

We have designed a measuring chamber optimized both for flow-through and “static” experiments. The cell hosts 14 mm-diameter quartz crystals with backside-reported electrodes fitting into a base which also provides the gold-coated spring contacts to the electrodes (Figure 2A).

Our top chambers fit precisely into the base and confine the solution under test by means of an O-ring with the very same diameter as the base O-ring, thus exerting a balanced pressure against this quartz and preventing cracks. The flow-through version has two 1 mm-diameter holes for inlet and outlet terminating to a pair of Luer^TM^ fittings for easy connection of the fluid system (Figure 2B).

The full assembly consists then of 3 parts—a PCB firmly anchored to the base, the base itself and the top part, which can be easily removed for inspection or to change the quartz sensor by means of hand-nuts. The whole parts as realized are visible in Figure 2C. 

The cell supplied allows, with a small modification, for work in both static and flow modes, ensuring the integrity of the crystal and easy contact with the gold electrodes, simply by placing the crystal on a rubber O-ring. In Figure 2C on the left, the cell (support, central) and the TOPs to be used for flow (FM, left) and static (SM, center) measurement modes are shown, respectively. The TOP SM piece has been kindly 3D printed in polylactic acid (PLA), by Dr. F.M. Vivaldi at the Department of Chemistry and Industrial Chemistry of the University of Pisa. The TOP SM is assembled with the support piece to originate the static mode measuring cell used in this work. This static cell has a central hole that allows for the direct addition of small volumes of solution (150 μL) with a micropipette and subsequent aspiration and washing of the chip surface. This mode allows us to work with small volumes of protein or nucleic acid solutions and/or small volumes of sample; this last cell was used here.

### 2.3. Spectrophotometric Analysis of PNE Film Polymerization

Two solutions of NE 2 mg/mL were obtained, in TRIS buffer with different ionic strengths, TRIS 10 and 50 mM, respectively. The 96-well polystyrene microtiter plates were used to evaluate polymer growth, and 100 μL of each solution were placed in the wells of the plate; their growth was evaluated at t = 0, t = 3 h, t = 6 h and t = 24 h. Measurements were performed in triplicate using the SPECTROstar Nano, a 96-well microtiter plate reader produced by BMG Labtech (Ortenberg, Germany)at a wavelength of 350 nm.

### 2.4. Synthesis of PNE MIP for IgG on QCM Quartz Crystal with Bare Gold Surface

The PNE MIP film was synthesized directly on a bare gold electrode of QCM sensor chips by the following effective and sustainable procedure, based on epitope imprinting [24]. A portion of the protein, i.e., the peptide ^439^KSLSLSPGK^447^ (acronym Fc CH3, MW = 916.08 g mol^−1^, purity > 95%) which belongs to the heavy chain of the IgG1 constant domain, was used here as the templating peptide; Fc CH3 has been previously selected [24,25] as the most performing peptide to selectively bind IgG and is hereafter referred to as the template, in the PNE-MIP synthesis. The peptide ^338^ISKAKGQP^345^ (acronym P338_L8, MW = 827.98 g mol^−1^, purity > 95%) also belongs to the heavy chain of the IgG1 constant domain and was used as the negative control.

The procedure includes the preparation of two solutions: (1) 800 μM solution of template peptide in water; (2) 4 mg/mL solution of NE monomer in TRIS-HCl buffer (20 mM pH 8.5) in which the solid is dissolved in buffer as late as possible since the self-polymerization reaction starts immediately. Equal volumes (100 μL) of the peptide and the monomer solutions are then deposited on the chip, for a total of 200 μL, to cover it entirely. The final concentrations are 2 mg/mL for the monomer and 400 μM for the peptide, respectively. The QCM crystal is placed in a humid chamber and left at 25 °C in an incubator for 5 h. Thereafter, the chip is removed and washed with 200 μL of water. Finally, 200 μL of passivation mixture is deposited on the surface, which is then reinserted into the humid chamber and left at 3 °C overnight (o.n.) for passivation, involving the catechol ring sites of the NE, not reacted during polymerization; to note, this step is crucial to minimize the non-specific adsorption from matrix components other than the target. The passivation mixture is composed of 11-mercapto-1-undecanol (MCU) and 6-mercapto-1-hexanol (MCH), both 1 mM in 10% ethanol. After the passivation step, the chip is washed with 200 μL of 20% EtOH to solubilize any unreacted thiols, and with 200 μL water solution. The activation of the PNE film deposited on gold substrates, aimed at removing the templating agent, was carried out through different washing steps. A volume of 200 μL of 5% (*v*/*v*) acetic acid was applied to the surface and subsequently removed, followed by the addition of 200 μL of deionized water. This washing cycle was repeated twice, resulting in a total of four alternating rinses with acetic acid and water. The chip was mounted in the measurement cell, and six sequential injections of 5% (*v*/*v*) acetic acid were performed, each lasting 30 s. This was followed by a 30 s injection of 0.005% (*w*/*v*) sodium dodecyl sulfate (SDS), and then an additional 30 s injection of 5% acetic acid. The SDS/acetic acid cycle was repeated once more to ensure thorough removal of the templating agent activation of the PNE film.

In this study, the static-mode cell top was used. Thus, the solutions were added manually with a micropipette. Sensorgrams were acquired with the WinQCM 3.0 software, and data analysis was performed using Origin V10, and Excel software. Calibration experiments were performed using a single-cycle kinetic (SCK) protocol, minimizing surface regeneration steps, and adding analyte solutions sequentially. For the standard addition method, for IgG quantification in human serum, the multiple-cycle kinetic (MCK) mode was applied, regenerating the quartz surface between different samples. This approach ensures baseline recovery between measurements and minimizes cumulative matrix effects associated with repeated exposure of the sensing surface to complex biological samples. The limit of detection (LOD) was calculated as 3σ/S, where σ is the standard deviation of the blank signal and S is the slope of the calibration curve. The limit of quantification (LOQ) was calculated as 10σ/S, where σ is the standard deviation of the blank signal and S is the slope of the calibration curve. The equilibrium dissociation constant (K_D_) was estimated by fitting the equilibrium frequency shifts (Δf) as a function of analyte concentration using the Langmuir adsorption model.

## 3. Results and Discussion

The aim of the work is the development of QCM-based affinity sensing based on molecularly imprinted biopolymers (MIPs), using bioinspired monomers present in nature and a new instrument WinQCM here developed. As analytical targets we first focused on IgG, recently explored by the research group, a protein target of clinical diagnostic interest [24,25]. To develop this green and low-cost sensor, we used NE as the monomer together with a peptide selected in the human IgG amino acidic (a.a.) sequence to obtain PNE-MIP films; the MIP is obtained by the epitope-imprinting approach, where the monomer polymerizes by autooxidation in the presence of the template peptide, here selected on the IgG Fc portion, which proved to be very efficient to bind whole IgG molecules [24,25].

The PNE polymer possesses key adhesive properties for the sensor modification, simplifying significantly the chip preparation and avoiding cumbersome and toxic use of hazardous chemicals [22,23]. From the sensing point of view, this approach with the PNE polymer speeds up the chip preparation, thanks to the autoxidative polymer properties, resulting in a very low-cost approach in terms of monomer usage (500 mg costs 255.00 euros) and worker exposure (the polymerization uses water as solvent) with no release of toxic byproducts into the environment.

First, the instrumentation performance of the newly developed WinQCM has been evaluated in terms of resolution, stability, and suitability for operation in liquid and low-Q conditions. Subsequently, the synthesis and characterization of the PNE-based imprinted films are discussed, followed by their application to the selective detection of human IgG in buffer and in human serum first, and further in serum as a proof of concept for sustainable, antibody-free affinity sensing by PNE-MIPs.

### 3.1. Analytical Performance of the WinQCM System

Baseline stability was assessed by monitoring the resonance frequency of a bare gold-coated quartz crystal immersed in phosphate-buffered saline (PBS, 10 mM, pH 7.4) under static conditions. The system exhibited a stable baseline with negligible drift over extended acquisition times, demonstrating its suitability for quantitative affinity measurements in aqueous media. The low instrumental noise and high temporal resolution allow for the detection of subtle mass variations associated with biomolecular recognition events.

The measuring chamber was specifically designed to minimize sample consumption while ensuring reliable electrical contact and mechanical stability of the quartz crystal. In static mode, a sample volume of approximately 150 µL is sufficient to fully cover the active electrode area, enabling analyses with limited amounts of biological samples. The chamber is also compatible with flow-through operation, providing versatility for both equilibrium-based and kinetic measurements.

Overall, the analytical performance of WinQCM (Table 1), combining sub-Hz frequency resolution, fast data acquisition, and robust operation under low-Q conditions, makes the system particularly well suited for affinity biosensing applications based on viscoelastic and biomimetic recognition layers, such as molecularly imprinted polymers. In addition, the compact design, low power consumption, and use of modular and partially biodegradable components align the instrument with the principles of Green Analytical Chemistry.

Under static conditions in PBS (10 mM, pH 7.4), the WinQCM system exhibited a low baseline noise, with a root mean square (RMS) frequency fluctuation below 0.05 Hz over a 10 min acquisition window. Baseline drift was negligible within the time scale of the experiments, remaining below 0.2 Hz h^−1^, thus enabling reliable detection of small frequency variations associated with biomolecular binding events. This level of stability is particularly relevant for affinity sensing in liquid media, where low-Q conditions typically limit frequency resolution.

The WinQCM system integrates a heterodyne-based frequency measurement architecture that enables deterministic evaluation of the quartz resonance frequency with sub-Hertz resolution. This capability was experimentally verified by applying controlled frequency perturbations and monitoring the system response, demonstrating a minimum resolvable frequency variation of 0.1 Hz (Figure S2). Such resolution is particularly advantageous for biosensing applications, as it allows reliable detection of the small frequency shifts associated with protein-binding events at low surface coverage.

The frequency resolution and baseline stability achieved with the WinQCM system are comparable to, or exceed, those reported for other compact and custom-built QCM platforms designed for operation in liquid media, where low Quality Factors typically limit sensitivity and long-term stability [11,18,29].

Based on these analytical characteristics, the WinQCM system was subsequently applied to affinity sensing experiments employing the PNE-BMIP film as biomimetic recognition layers, targeting IgG. The combination of high frequency resolution, rapid acquisition rate, and stable operation under low-Q conditions is particularly advantageous for monitoring protein-binding events on viscoelastic polymeric interfaces, as demonstrated in the following sections.

### 3.2. PNE Polymerization and Film Formation

#### 3.2.1. Selection of the Template Peptide and Epitope-Imprinting Strategy

To obtain MIPs using an epitope approach for the recognition of IgG1 immunoglobulins, short peptide sequences were employed as templates to generate selective binding cavities capable of discriminating the target IgG1 within the pool of immunoglobulin isotypes and classes (IgA, IgG, IgM, IgE) physiologically present in human serum [27]. Among Immunoglobulin G subclasses, four isoforms (IgG1, IgG2, IgG3, and IgG4) are distinguished in decreasing order of abundance and share a high degree of homology in the primary structure of their heavy chains (approximately 90%) [25].

The identification of suitable epitopes for imprinting was therefore based on the evaluation of sequence differences within the crystallizable (Fc) region, with the aim of achieving selective recognition of IgG1 while maintaining affinity toward the IgG class as a whole. In this context, the Fc CH3 peptide was selected as the imprinting template as it exhibits sequence variability among immunoglobulin subclasses and is structurally exposed on the native protein. Owing to these features, the Fc CH3 sequence has been previously reported as an effective epitope for selective IgG recognition in biomimetic sensing platforms [24,25].

The epitope-based imprinting strategy enables the formation of well-defined and accessible recognition sites while avoiding the steric hindrance and conformational complexity associated with full-protein imprinting. In the present work, the suitability of the Fc CH3 peptide as a template for PNE-based molecular imprinting is experimentally validated by the binding and selectivity results discussed in the following sections. Notably, the use of a short Fc-derived epitope as the template (rather than the full protein) favors the formation of accessible recognition sites while avoiding steric hindrance and conformational complexity; at the same time, it yields interactions sufficiently strong for selective IgG capture but still compatible with regeneration in limited-reuse assay formats [24,25]. The PNE-BMIP film was then examined by UV–Vis spectrophotometry to monitor the polymer formation process and to evaluate the influence of the buffer conditions.

#### 3.2.2. UV–Vis Monitoring of PNE Polymerization and Film Adhesion

The formation of PNE films was investigated by UV–Vis spectrophotometry to monitor the polymerization kinetics and evaluate the influence of buffer ionic strength on its growth and adhesion on polystyrene microwell plates. NE undergoes spontaneous oxidative polymerization in mildly alkaline aqueous solutions, leading to the formation of an adhesive PNE layer. The spontaneous oxidative polymerization of NE under mildly alkaline conditions and the strong adhesive properties of the resulting PNE films are well documented in the literature and have been successfully exploited for surface functionalization and biosensing applications [22,23]. The presence of Tris buffer is known to promote cross-linking and film formation, acting as an adjuvant for the polymerization process. The promoting effect of Tris buffer on catecholamine polymerization and cross-linking has been previously reported and is attributed to its role in stabilizing reactive intermediates during oxidative polymerization [23].

**Figure 3:**
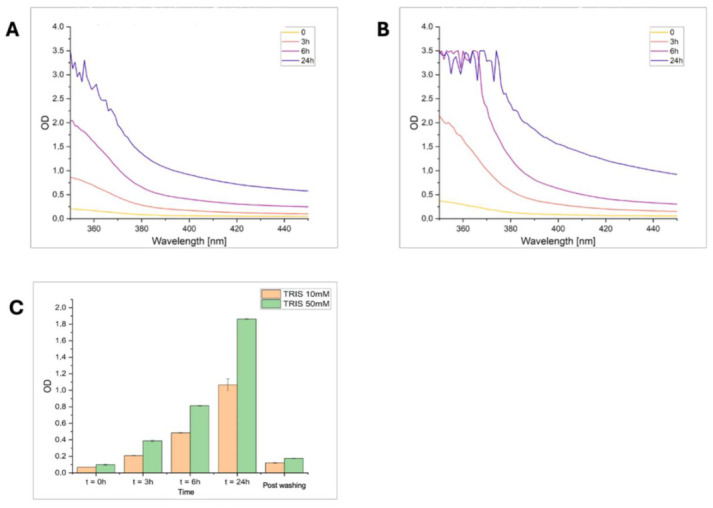
UV–Vis absorption spectra of PNE polymerization recorded at different incubation times in Tris-HCl buffer (10 and 50 mM, pH 8.5) λ = 350–450. (**A**) In 10 mM TRIS; (**B**) in 50 mM TRIS. (**C**) Comparison of the PNE absorption profile in Tris 10 and 50 mM, respectively, recorded @390 nm and at different times.

Polymer growth was monitored over time by recording UV–Vis absorption spectra of NE solutions (2 mg mL^−1^) prepared in Tris-HCl buffer at two different ionic strengths (10 and 50 mM, pH 8.5) (Figure 3A,B). The evolution of the absorption profile in the 350–450 nm range was followed at selected time points up to 24 h (Figure 3C). In both conditions, a progressive increase in absorbance was observed, indicating the formation of the PNE polymer. However, a markedly faster growth rate and higher final absorbance were obtained in 50 mM Tris-HCl, highlighting the strong influence of buffer composition on polymerization efficiency.

To evaluate the amount of PNE polymer remaining adhered to the microwell surface after the washing step, the residual absorbance at 390 nm was measured. This post-washing absorbance reflects the PNE still bound to the surface and thus the adhesion of the polymer to the substrate, a key feature enabling direct surface functionalization without additional anchoring chemistries. These results guided the selection of the polymerization conditions adopted for preparing PNE-based imprinted films on gold-coated QCM crystals.

### 3.3. IgG Binding on PNE-MIP/QCM in Buffer

The affinity binding performance of the PNE-MIP films toward Human Immunoglobulin G (IgG) was evaluated using the WinQCM system under static conditions in phosphate-buffered saline (PBS, 10 mM, pH 7.4). All measurements were carried out using quartz crystals functionalized with PNE-BMIP films synthesized directly on the gold electrode surface, as described in Section 2, using a single-frequency QCM (non-dissipation mode).

Initially, to better elucidate the nature of the QCM response during IgG binding, both frequency (Δf) and dissipation (ΔD) signals were monitored on the MIP surface, and a representative graphical representation of ΔD vs. Δf analysis is reported in Figure S3. The observed frequency shifts are accompanied by small but measurable variations in dissipation (ΔD on the order of 10^−6^–10^−5^), indicating that the response includes a slight viscoelastic contribution associated with the PNE-MIP layer, in agreement with previous reports on MIP-based QCM systems [30]. However, the relatively low ΔD values suggest that the formed layer behaves as a relatively rigid film [31]. Under these conditions, the sensor response can be considered predominantly mass-related but still includes non-negligible viscoelastic contributions, although not strictly within the Sauerbrey regime [32]. In liquid-phase conditions, the QCM response is more appropriately described by the Kanazawa and Gordon equation (Equation (1)) [6], considering that the contact of a QCM sensor with a liquid causes changes in oscillation that are proportional to the absolute viscosity (*ηₗ*) and density (*ρₗ*) of the liquid and of the quartz (*η_q_*), and the shear modulus (*μ_q_*) respectively.

(1) $$∆f=-2f_{0}^{3/2}\sqrt{\frac{\eta ₗ\;\rho ₗ}{\pi \rho_{q}\mu_{q}}}$$

Because the measurement cycle involves both measurement and washing solutions, in order to minimize these effects, all measurements were performed in the same buffer solution, ensuring constant viscosity and density during both binding and washing steps.

**Figure 4:**
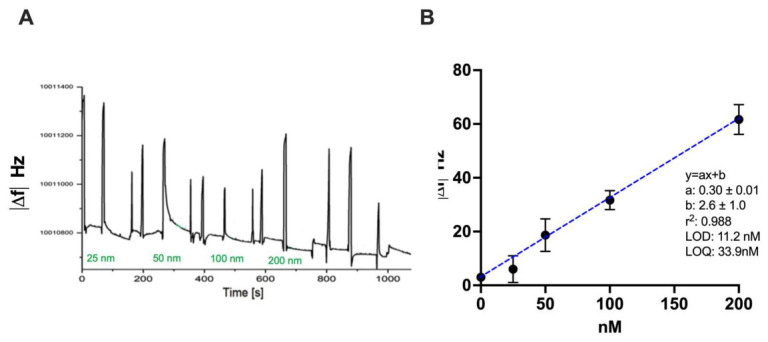
(**A**) QCM sensorgram showing the frequency shift (Δf) recorded during single-cycle kinetic (SCK) measurements upon successive addition of IgG solutions (0–200 nM) in PBS (10 mM, pH 7.4). Measurements were performed in static mode by depositing 150 μL of solution onto the sensor surface (interaction time 90 s), followed by 30 s buffer washing. Baseline stabilization was achieved by depositing buffer for 60 s prior to each measurement. The spikes observed in the signal correspond to sample removal and addition; (**B**) calibration curve obtained by plotting the absolute frequency shift (|Δf|) as a function of IgG concentration (n = 3).

Protein recognition experiments were performed by successive exposure of the sensor surface to increasing concentrations of IgG using a single-cycle kinetic (SCK) approach, thereby minimizing regeneration steps. Analyte solutions (150 µL) were added to the cell and allowed to interact with the sensing layer for 90 s, followed by a 30 s washing step with buffer to remove unbound species. The solution was then replaced with buffer (150 µL) to allow baseline stabilization (60 s) before proceeding to the next analyte solution. The recorded sensorgrams show a concentration-dependent decrease in resonance frequency upon IgG binding, consistent with mass accumulation on the quartz surface. (Figure 4A) The absence of significant baseline drift during buffer rinsing confirms the stability of the PNE-MIP layer. The binding response increased progressively with IgG concentration, indicating the accessibility and functionality of the imprinted recognition sites, up to a plateau. During IgG binding experiments, only minor variations in the motional resistance, and consequently in the Quality Factor (Q), were observed compared to the corresponding frequency shifts. This behavior indicates that the QCM response is predominantly driven by mass loading rather than by viscoelastic damping of the PNE-MIP layer, supporting the validity of frequency-based quantification.

A calibration curve was constructed by plotting the absolute value of the frequency shift (|Δf|) as a function of IgG concentration in the range 0–400 nM (Figure 4B). Each concentration was measured in triplicate. The IgG calibration curve showed a good linear relationship, described by the equation y = 0.30x + 2.6 (slope: 0.30 ± 0.01; intercept 2.6 ± 1.0). The regression coefficient (R^2^ = 0.988) confirms the good linearity of the method within the investigated concentration range. The analytical sensitivity of the system was assessed by calculating the limit of detection (LOD = 11.2 nM) and the limit of quantification (LOQ = 33.9 nM) using the conventional criteria based on the ratio between the standard deviation of the response and the slope of the calibration curve. The repeatability of the measurements, expressed as the average relative standard deviation (%RSD_av_ = 10.9), indicates acceptable precision of the QCM measurements. 

The dissociation constant (K_D_) was estimated by nonlinear fitting of the binding isotherm according to the Langmuir adsorption model. K_D_ falls in the sub-micromolar range, consistent with affinity values typically reported for epitope-imprinted polymer systems and is consistent with values previously observed for IgG recognition using biomimetic sensing platforms [25,33,34,35]. Although higher than those observed for antibody-based assays, this affinity is sufficient for quantitative determination of IgG in diluted serum samples and reflects the synthetic and biomimetic nature of the recognition layer.

These results demonstrate that the PNE-BMIP/QCM platform enables reproducible and quantitative detection of IgG. The combination of a stable biomimetic recognition layer and the high frequency resolution of the WinQCM system allows monitoring of protein-binding events, providing a solid basis for subsequent selectivity studies and application in complex biological matrices, see Table 2.

### 3.4. Selectivity and Imprinting Efficiency

**Figure 5:**
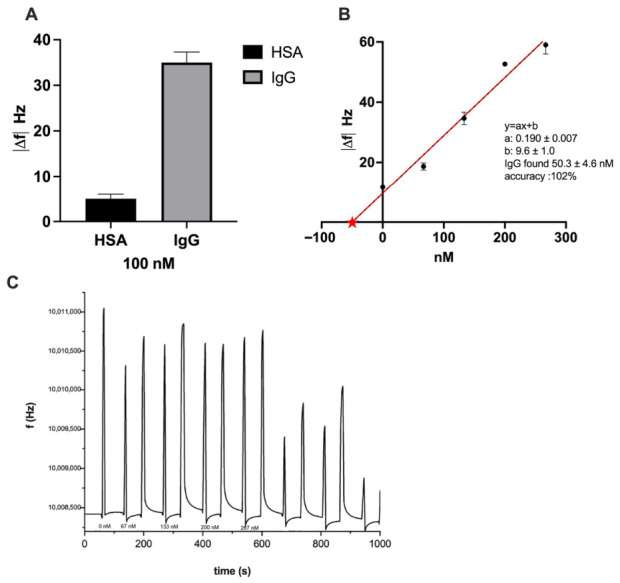
(**A**) Selectivity evaluation of the PNE-BMIP/QCM sensor. Absolute frequency shifts (|Δf|) recorded for IgG and HSA at 100 nM. Data are reported as mean ± standard deviation (n = 3). (**B**) Standard addition plot for IgG determination (0–200 nM) in 1:1000 diluted human serum. The extrapolated concentration corresponds to 50.3 ± 4.6 nM in the diluted sample, equivalent to approximately 7.5 mg mL^−1^ in the original serum. (**C**) QCM sensorgram showing the frequency shift (Δf) recorded during multi-cycle kinetic (MCK) measurements upon standard addition of IgG solutions (0–200 nM) in diluted human serum (1:1000 in PBS). Measurements were performed in static mode by depositing 150 μL of sample onto the PNE-BMIP-modified quartz crystal, allowing interaction for 90 s, followed by 30 s washing with running buffer. Baseline stabilization was achieved by depositing buffer for 60 s prior to each measurement. Signal spikes correspond to sample removal and addition.

The selectivity of the PNE-based molecularly imprinted polymer toward IgG was evaluated by comparing its binding response to that obtained for human serum albumin (HSA), a highly abundant protein in human serum commonly used as a competing species in selectivity studies. Experiments were performed under the same experimental conditions adopted for IgG binding, using equimolar concentrations of IgG and HSA to enable a meaningful comparison of affinity behavior (Figure 5A).

Since QCM is inherently a mass-sensitive technique, the raw frequency shifts obtained for IgG and HSA 100 nM were with respect to the molecular weight of the two proteins to account for their different masses (approximately 150 kDa for IgG and 66 kDa for HSA). The responses were approximately normalized to account for differences in molecular mass, although QCM signals may also be influenced by hydration and viscoelastic effects. This normalization allows the comparison to reflect binding selectivity rather than purely gravimetric effects. Accordingly, the selectivity factor (α) was introduced as a comparative metric to summarize the normalized discrimination between target and non-target proteins in a mass-sensitive transduction scheme, facilitating comparison with literature values reported for protein-imprinted polymer receptors. The selectivity factor (α) was calculated as the ratio between the normalized frequency responses obtained for the target protein (IgG) and for the competing protein (HSA).

The PNE-MIP-coated sensors exhibited a significantly higher normalized frequency shift for IgG compared to HSA (Figure 5A), indicating preferential recognition of the target protein. Although a certain level of non-specific adsorption was observed for HSA, as expected for protein interactions on polymeric surfaces, the response toward IgG was markedly enhanced, confirming the effectiveness of the imprinting process. The imprinting efficiency was quantitatively assessed by calculating the selectivity factor (α), defined as the ratio between the QCM response obtained for the target analyte (IgG) and that measured for the competing protein (HSA). The resulting α value of 3.1 demonstrates a clear preference of the PNE-MIP film for IgG over HSA and is consistent with values reported for protein-imprinted polymer systems based on epitope-imprinting strategies.

Selectivity factors in the range of 2–5 are commonly reported for protein-imprinted polymer systems, particularly when epitope-imprinting strategies are employed to discriminate between structurally related proteins in complex environments [33]. Overall, these results confirm that the PNE-based imprinting approach provides selective recognition of IgG, supporting the suitability of the developed PNE-BMIP/QCM platform for application in complex biological matrices where non-specific protein adsorption represents a critical challenge.

### 3.5. IgG Determination in Human Serum

The applicability of the PNE-BMIP/QCM platform to complex biological matrices was evaluated by determining IgG in human serum (HS). Due to the high physiological concentration of IgG, HS samples were diluted 1:1000 in PBS (10 mM, pH 7.4) to keep the analyte concentration within the dynamic range of the assay. Quantitative analysis was performed by the standard addition method, which is well suited to compensate for matrix effects in complex samples.

Aliquots of diluted HS were spiked with increasing concentrations of IgG and analyzed using a multiple-cycle kinetic (MCK), by regeneration between the sample injection. This approach was well suited for complex matrices; it ensures baseline recovery, minimizing cumulative matrix effects associated with repeated exposure HS. Each measurement was performed in static mode by depositing 150 μL of standard solution onto the PNE-BMIP-modified quartz crystal surface and allowing interaction for 90 s. The stabilized frequency shift (Δf) was then recorded, followed by a 30 s washing step with buffer. Signal stability was monitored after 60 s of buffer deposition to allow baseline stabilization. The sensor surface was regenerated between measurements by short exposures to acidic and surfactant solutions (5% *v*/*v* acetic acid and dilute SDS; see Section 2) and by alternating depositions of 50 mM HCl and 0.05% SDS until the baseline signal was restored. No significant loss of response was observed after repeated regeneration cycles (n = 5).

The resulting standard addition plot exhibited a linear response within the investigated concentration range, enabling the determination of IgG in the diluted serum sample (Figure 5B; Table 3) and the relative sensorgram is reported in Figure 5C. From the linear regression analysis, an IgG concentration of 50.3 ± 4.6 nM was obtained for the 1:1000 diluted serum sample, corresponding to approximately 7.5 mg mL^−1^ in the undiluted serum. The determination of IgG concentration in diluted human serum samples using the PNE-BMIP/QCM platform, combined with the standard addition method, demonstrated good agreement with the reference nephelometric analysis. As shown in Table 3, the measured IgG concentration (50.3 ± 4.6 nM) is very close to the value obtained by the reference method (49.3 nM), resulting in an accuracy of 102.0%. The low standard deviation (n = 3) suggests good repeatability (%RSD_av_ = 4.6%) of the measurements, confirming the reliability of the proposed sensing platform in this real matrix. These results highlight the capability of the PNE-BMIP/QCM system to accurately quantify IgG in complex biological matrices such as human serum, without significant matrix interference. Overall, the method proves to be a valid alternative to conventional nephelometric techniques, offering comparable accuracy with the potential advantages of reduced sample preparation and rapid analysis.

Overall, these results highlight the reliability and analytical suitability of the developed QCM sensor for quantitative IgG determination.

The obtained results demonstrate the capability of the PNE-BMIP/QCM system to detect and quantify IgG in real biological samples, highlighting its potential as a sustainable, antibody-free analytical tool for protein determination in complex media. These results confirm the applicability of the proposed platform to real biological samples.

## 4. Conclusions

In this work, a novel compact QCM instrumentation (WinQCM) has been developed and analytically validated for affinity biosensing applications in liquid media, including biological samples with application in clinical diagnostic. The system combines high frequency resolution, rapid data acquisition, and stable operation under low-Q conditions, enabling reliable monitoring of biomolecular interactions on viscoelastic recognition layers. These features make WinQCM particularly suitable for biosensing applications based on polymeric and biomimetic interfaces.

As a proof of concept, the WinQCM system was coupled with sustainable molecularly imprinted polynorepinephrine (PNE-BMIP) films as, antibody-free recognition elements. The PNE-BMIP layers were synthesized directly on gold-coated quartz crystals through a simple and environmentally benign procedure, providing stable and adhesive films suitable for QCM measurements. The resulting PNE-BMIP/QCM platform enabled IgG quantitative determination in human serum using a standard addition approach.

Overall, this study demonstrates that the integration of compact, energy-efficient WinQCM instrumentation with bioinspired molecularly imprinted polymers represents a promising strategy for the development of sustainable affinity biosensors. The proposed approach expands the portfolio of transducers compatible with PNE-based imprinted receptors, so far applied to optical sensing (Bilayer Interpherometry, BLI, Surface Plasmon Resonance, SPR and spectrophotometric assays) [24,25] and opens new perspectives for antibody-free protein sensing in clinical and bioanalytical applications. Future work will focus on extending the WinQCM platform to multiplexed detection and integration with microfluidic systems for automated biosensing applications.:

## Figures and Tables

**Table 1:** Analytical specifications of the WinQCM system.

Parameter	WinQCM (This Work)	Relevance for Biosensing
Quartz type	AT-cut	Standard for liquid-phase QCM
Fundamental frequency	10 MHz	High mass sensitivity
Frequency resolution	~0.01 Hz	Sub-Hz gravimetric detection
Temporal resolution	0.1 s	Real-time kinetic monitoring
Q-factor measurement	Ring-down method	Direct dissipation assessment
Measurement modes	Static and flow-through	Versatile assay formats
Minimum sample volume (static)	~150 µL	Reduced sample consumption
Chamber materials	Polymeric/PLA components	Green analytical design
Power consumption	Low (portable prototype)	Energy-efficient operation

**Table 2:** Apparent binding parameters of IgG binding data, including dissociation constant (KD) and maximum frequency response (R_max). Values are reported as mean ± standard deviation (n = 3).

Target	KD [nM]	Rmax [Hz]
IgG	426.1 ± 152.1	174.7 ± 42.1

**Table 3:** Determination of IgG concentration in diluted human serum samples using the PNE-BMIP/QCM platform and standard addition method. The results were compared with reference nephelometric analysis. Values are reported as mean ± standard deviation (n = 3).

Sample	Dilution Factor	IgG Found (nM)	Reference Method (nM)	Accuracy (%)
Human serum	1:1000	50.3 ± 4.6	49.3	102.0%

## Data Availability

Data is contained within the article or Supplementary Material.

## Supplementary Materials

The following supporting information can be downloaded at: https://www.mdpi.com/article/10.3390/s26102985/s1, Figure S1: (A) Left: schematic representation of a generic feedback-controlled oscillator implementing a 180° phase rotation principle. Right: electrical equivalent circuit of a quartz crystal (Butterworth–Van Dyke model), where C_0_ represents the static capacitance of the electrodes, R accounts for energy losses, L is proportional to the oscillating mass, and C_1_ reflects the mechanical compliance of the crystal (how much the crystal deforms mechanically when a force or electric field, as in our case, is applied). (B) Electrical insulation scheme of the quartz crystal from the system ground reference (GND). Pins QA and QB allow electrical connection to external electrochemical instrumentation, enabling combined electrochemical and QCM measurements without affecting frequency and dissipation readout. (C) Schematic representation of the high-resolution frequency readout mechanism implemented in the WinQCM system. The quartz oscillation signal is mixed with a stable reference frequency, and the resulting difference signal is processed using a dual-timer acquisition strategy to achieve sub-Hz frequency resolution. Figure S2: Experimental verification of the sub-Hertz frequency resolution of the WinQCM system. Controlled frequency perturbations of 0.1 Hz were applied using an external signal generator. The system reliably resolves sub-Hertz frequency variations under the same acquisition conditions employed for biosensing experiments, demonstrating its suitability for detecting small frequency shifts associated with protein binding events. Figure S3: Simultaneous frequency (Δf) and dissipation (ΔD) monitoring during IgG binding to the MIP surface. The relatively small variations in ΔD (10^−6^–10^−5^) compared to Δf indicate a predominantly mass-dominated response with limited viscoelastic contributions, consistent with the behavior of thin and relatively rigid sensing layers.

## Abbreviations

The following abbreviations are used in this manuscript:

QCM	Quartz Crystal Microbalance
QCM-D	Quartz Crystal Microbalance with Dissipation monitoring
MIP	Molecularly Imprinted Polymer
PNE	Polynorepinephrine
PNE-MIP	Polynorepinephrine-based Molecularly Imprinted Polymer
IgG	Immunoglobulin G
HSA	Human Serum Albumin
NE	Norepinephrine
TRIS	Tris(hydroxymethyl)aminomethane
PBS	Phosphate-Buffered Saline
SDS	Sodium Dodecyl Sulfate
MCU	11-Mercapto-1-undecanol
MCH	6-Mercapto-1-hexanol
SCK	Single-Cycle Kinetic
MCK	Multiple-Cycle Kinetic
LOD	Limit of Detection
LOQ	Limit of Quantitation
KD	Equilibrium Dissociation Constant
RMS	Root Mean Square
AGC	Automatic Gain Controller
ADC	Analog-to-Digital Converter
CPU	Central Processing Unit
PCB	Printed Circuit Board
PLA	Polylactic Acid
