# Supplementary material for: Development of an Improved QCM-D Instrumentation for Affinity Sensing by Bioinspired Molecular-Imprinted Polymers (MIP) for IgG Detection in Serum
Source: Sensors (Basel). 2026 May 9;26(10):2985. doi: 10.3390/s26102985 (PMC13210962; doi:10.3390/s26102985)
Supplement: Supplementary file 1 [file sensors-26-02985-s001.zip › sensors-4238561-supplementary.pdf]

SUPPORTING INFORMATION FOR

Development of an improved QCM-D instrumentation for af-finity sensing by bioinspired Molecular Imprinted Polymers (MIP) for IgG detection in serum

Doretta Cuffaro1, Lucia Bonasera1, Elisa Nuti1, Riccardo Galletti2, Manuela Adami2, Marco Sartore2, and Maria Minunni1**

1 Department of Pharmacy, University of Pisa, 56125 Pisa, Italy; lucia.bonasera@edu.unito.it (L.B.); elisa.nuti@unipi.it (E.N.)

2 ElbaTech Srl, 57030 Marciana, LI, Italy; galletti@elbatech.com (R.G.); adami@elbatech.com (M.A.); sartore@elbaetch.com (M.S.)

* Correspondence: doretta.cuffaro@unipi.it (D.C.); maria.minunni@unipi.it (M.M.)

Figures of Contents

I. Figures S1-S4

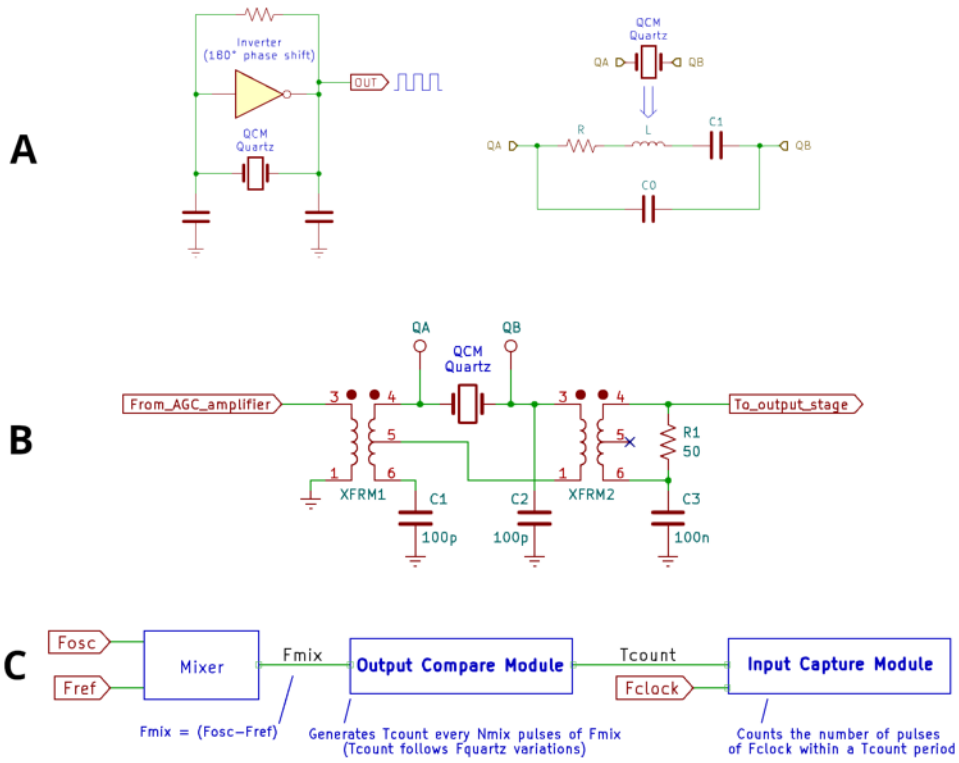

Figure S1. (A) Left: schematic representation of a generic feedback-controlled oscillator implementing a 180° phase rotation principle. Right: electrical equivalent circuit of a quartz crystal (Butterworth–Van Dyke model), where C_0 represents the static capacitance of the electrodes, R accounts for energy losses, L is proportional to the oscillating mass, and C_1 reflects the mechanical compliance of the crystal (how much the crystal deforms mechanically when a force or electric field, as in our case, is applied). (B) Electrical insulation scheme of the quartz crystal from the system ground reference (GND). Pins QA and QB allow electrical connection to external electrochemical instrumentation, enabling combined electrochemical and QCM measurements without affecting frequency and dissipation readout. (C) Schematic representation of the high-resolution frequency readout mechanism implemented in the WinQCM system. The quartz oscillation signal is mixed with a stable reference frequency, and the resulting difference signal is processed using a dual-timer acquisition strategy to achieve sub-Hz frequency resolution.

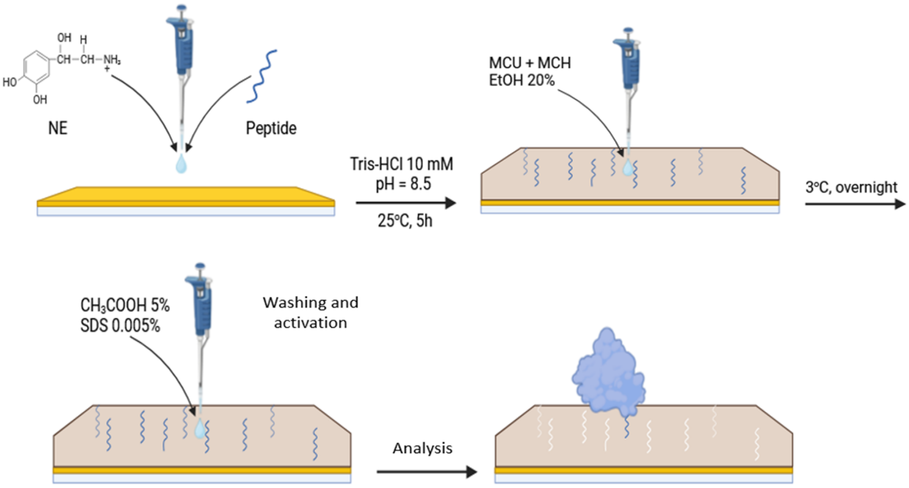

Figure S2. Synthetic procedure for the synthesis of PNE MIP film. (Biorender.com)

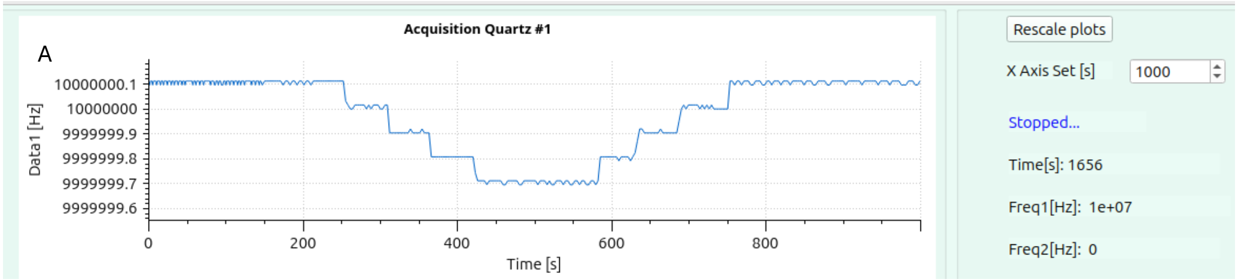

Figure S3. Experimental verification of the sub-Hertz frequency resolution of the WinQCM system. Controlled frequency perturbations of 0.1 Hz were applied using an external signal generator. The system reliably resolves sub-Hertz frequency variations under the same acquisition conditions employed for biosensing experiments, demonstrating its suitability for detecting small frequency shifts associated with protein binding events.

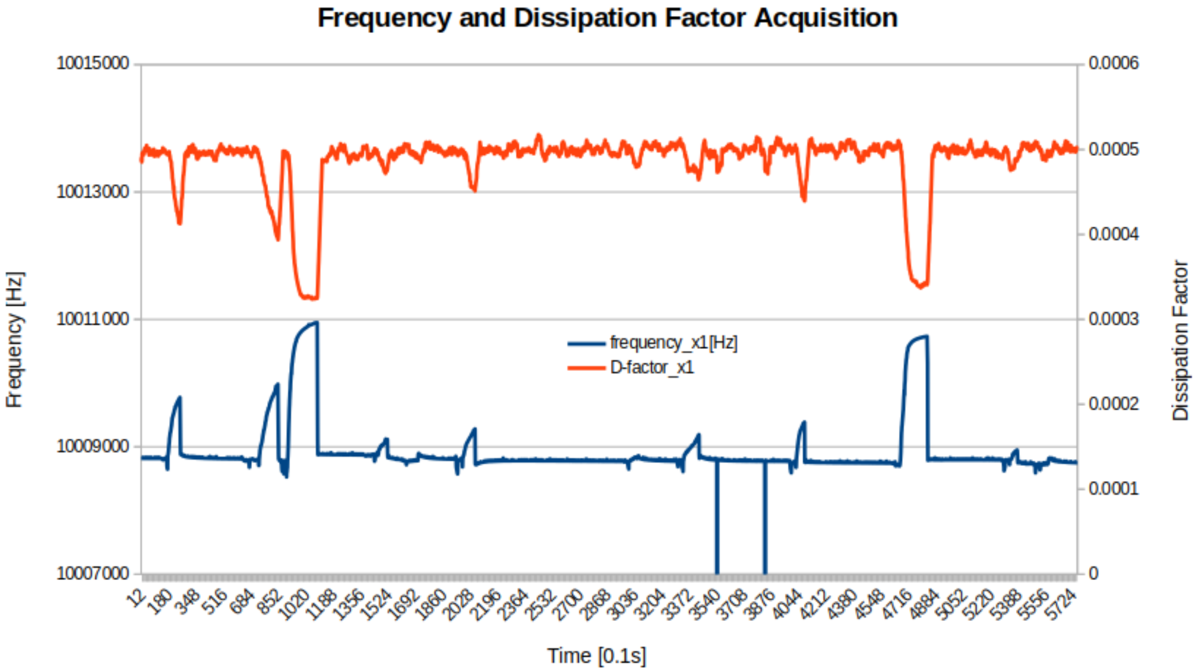

Figure S4. Simultaneous frequency (Δf) and dissipation (ΔD) monitoring during IgG binding to the MIP surface. The relatively small variations in ΔD (10^{-6}–10^{-5}) compared to Δf indicate a predominantly mass-dominated response with limited viscoelastic contributions, consistent with the behavior of thin and relatively rigid sensing layers
